# Is the 4-factor model of symptomology equivalent across bipolar disorder subtypes?

**DOI:** 10.1186/s40345-021-00229-1

**Published:** 2021-08-02

**Authors:** Norm O’Rourke, Andrew Sixsmith, Tal Michael, Yaacov G. Bachner

**Affiliations:** 1 Department of Public Health and Multidisciplinary Center for Research On Aging, Ben-Gurion University of the Negev, P.O. Box 653, 8410501 Be’er Sheva, Israel; 2 STAR Institute, Simon Fraser University, Vancouver, BC Canada; 3 School of Public Health, Faculty of Health Sciences, Ben-Gurion University of the Negev, Be’er Sheva, Israel

**Keywords:** Affrontive symptoms, BDS_x_, Bipolar disorder, BD subtypes, Invariance analyses, Scale validation

## Abstract

**Background:**

Research with the BDS_x_ (Bipolar Disorder Symptom Scale) suggests a 4-factor structure of responses: two depression (cognitive, somatic) and two hypo/mania factors (elation/loss of insight, affrontive symptoms). The two depression and two hypo/mania factors are correlated; and affrontive symptoms of hypo/mania (e.g., furious, disgusted, argumentative) are positively correlated with both depression factors suggesting pathways for mixed symptom presentation. This grouping of affrontive symptoms of hypo/mania organically emerged in exploratory research and has subsequently been supported in confirmatory analyses between samples and over time. The BDS_x_ has been clinically validated with BD outpatients.

**Results:**

Over 19 days, we recruited an international sample of 784 adults with BD using micro-targeted, social media advertising (M  =  44.48 years, range 18–82). All participants indicated that they had BD (subtype, if known) and had been diagnosed with BD (month, year). This sample size was sufficient to confirm the 4-factor model across subtypes and compare the three (BD I, BD II, BD NOS). Responses to 19 of 20 BDS_x_ items were psychometrically consistent across BD subtypes. Only responses to the ‘hopeless’ item were significantly higher for those with BD II.

**Conclusions:**

When comparing models, it appears that affrontive symptoms are significantly and uniformly associated with hypo/mania and both depression factors across subtypes. In contrast to BD diagnostic criteria, this suggests that affrontive symptoms are central to the clinical presentation of hypo/mania and mixed symptomology across BD subtypes.

## Background

Despite the efficacy of pharmacotherapy (Osher et al. 2010), the long-term course of bipolar disorder (BD) is characterized by frequent relapse, often with residual or subthreshold symptoms (Samalin et al. 2016; Treuer and Tohen 2010). BD is a leading cause of global disability (Catala-Lopez et al. 2013). Population growth and aging are further contributing to BD burden (Ferrari et al. 2016).

### Bipolar disorder subtypes

Those with BD I (previously, manic-depressive disorder; Phillips and Kupfer 2013) experience both episodes of major depression and mania with psychosis or loss of contact with reality (e.g., perceived special abilities). The negative effects of mania can endure long after (e.g., severed relationships, debts, exhausted savings).

Those with BD II also experience major depression, but more limited hypomania as opposed to full mania (Serretti and Olgiati 2005). Similar to BD I, those with BD II are euthymic most of the time (Saunders and Goodwin 2010) yet they may experience more episodes of depression, of longer duration and greater severity compared to BD I (Tondo et al. 2017).

The symptoms of those with BD not otherwise specified (BD NOS) do not fit cleanly within the criteria or other subtypes (Towbin et al. 2013). BD NOS is a catch-all category more than a defined subtype. The term, unspecified bipolar disorder is today preferred (APA; American Psychiatric Association 2013).

Those with cyclothymia do not meet criteria for either BD I or II (Van Meter et al. 2012); however, subclinical symptoms can extend over years. Cyclothymia may be the most prevalent BD subtype though the least frequently diagnosed (Van Meter et al. 2017); some, however, question whether if cyclothymia is in fact a distinct disorder (Parens and Johnston 2010).

### 4-factor model of BD symptomology

For the BADAS (Bipolar Affective Disorder and older Adults) Study, a sample of 1010 adults with BD were asked to indicate the extent to which 114 mood and symptom adjectives corresponded to how felt at that moment (O’Rourke et al. 2018). This pool of items was compiled by an expert team of clinicians and BD researchers. A 4-factor solution emerged from separate exploratory analyses (independent samples), confirmed over time (O’Rourke et al. 2016) and across age groups (O’Rourke et al. 2018). Each of these 4 factors are directly and indirectly related to quality of life with BD (O’Rourke et al. 2021).

**Fig. 1:**
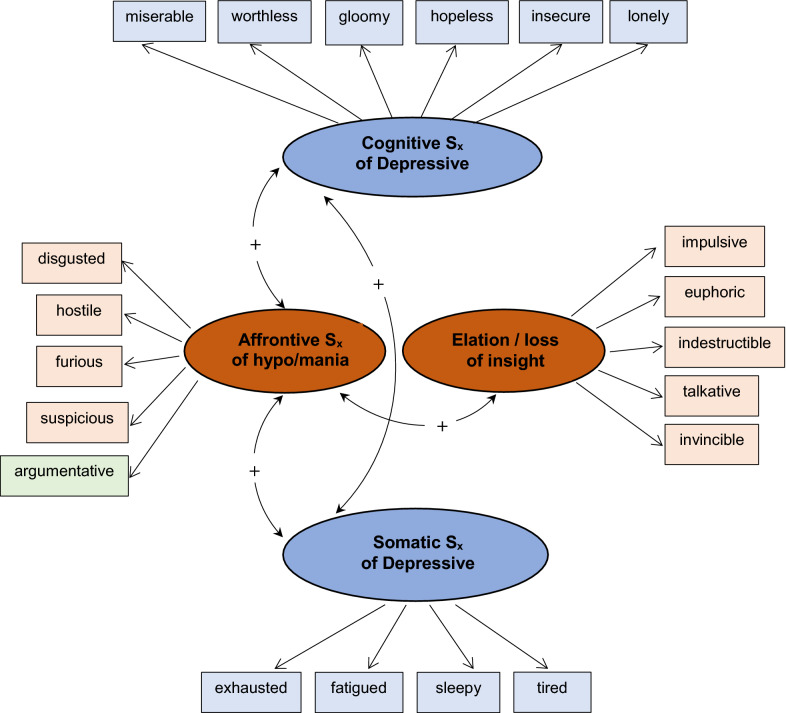
4-factor model of BD symptomology

This 4-factor model is composed of two correlated depression factors (cognitive, somatic symptoms) and two correlated hypo/mania factors (affrontive symptoms, elation/loss of insight). Affrontive symptoms of hypo/mania are also correlated with both depression factors suggestive of a mixed-symptom clinical presentation (American Psychiatric Association 2013). These symptoms reflect a confrontational, interpersonal expression of BD symptomology. See Fig. 1.

### Affrontive symptoms of hypo/mania

Recent research with BD participants indicates that affrontive symptoms of hypo/mania are correlated with negative partner mood (O’Rourke and King 2019), more so than other BDS_x_ factors. However, this effect is not statistically significant when couples are physically apart, only when together (i.e., same GPS coordinates), supporting the construct validity of this hostile, interpersonal cluster of symptoms. By contract, the traditional elation/loss of insight factor, more consistent with euphoric mania, appears to have little impact on partner mood (O’Rourke and King 2019).

Another possibility is that affrontive symptoms are not hypo/mania at all, but instead agitated depression. Irritability and anger outbursts occur under both definitions, but there is an interpersonal aspect to affrontive symptoms absent from current understanding of agitated depression. And as noted by Serra et al. (2019), agitation co-occurs with depression not hypo/mania, in particular, decreased libido, poor concentration, and suicidal ideation. With agitated depression, patients sometimes describe an underlying sense of anxiety or unease; with affrontive symptoms, the driving emotion is anger, often rage.

### Impact of BD on others

It is well documented that BD negatively affects others (Maskill et al. 2010; Maji et al. 2012; Perlick et al. 2008; Srivastava et al. 2010). Symptoms of depression appear to have greater impact that hypo/mania (Perlick et al. 1999; O’Rourke and King 2019) and suicidal ideation appears to impact friends and family most adversely (Chessick et al. 2009).

Despite the apparent significance of these interpersonal symptoms, discussion is limited in the research literature and largely absent from diagnostic criteria (i.e., near exclusive focus on individual, not interpersonal behavior). Affrontive symptoms may qualify as atypical features as DSM 5 lists interpersonal reaction sensitivity as one example (previously interpersonal rejection sensitivity; Peng et al. 2016). Yet affrontive symptoms are proactive not reactive (e.g., suspicious, hostile, argumentative).

For this study, we set out to independently replicate the 4-factor model across BD subtypes (BD I, BD II, BD NOS), assuming that affrontive symptoms would emerge with each. Moreover, we assumed that this affrontive factor would be correlated with elation/loss of insight and both depression factors (cognitive and somatic symptoms), suggesting that these affrontive symptoms are consistent across BD subtypes, and core to the clinical presentation of hypo/mania.

## Methods

### Participant recruitment

We recruited a global sample of 784 English-speaking adults: 218 with BD I, 277 with BD II and 289 with BD NOS. Responses from 37 respondents with cyclothymia were set aside as too few for latent variable analyses (less than 5%). This study was undertaken with the approval of the Research Ethics Board at Simon Fraser University, Burnaby (BC), Canada.

Data were collected over 19 days using social media advertising micro-targeted to Facebook users with BD. Participants were drawn from a global population of 6.2 million English-speaking, adult Facebook users with ‘bipolar disorder interests’ (e.g., members of online BD support networks). To expediate data collection, one randomly selected participant (in Ireland) received a $500 lottery prize.

As described elsewhere more thoroughly (King et al. 2014), machine-generated algorithms calculated by social media platforms are unique not so much for their sensitivity but specificity (i.e., exclusion of those who do not have BD). Persons recruited via Facebook are unlikely representative of the population but we can be confident these are persons with BD because only persons with BD received advertisements.

### Validity check

To corroborate that participants were in fact persons with BD, they were asked to list all prescribed BD medications. Most specified 1+  mood stabilizer (58.9%) and 1+  antidepressant (64.4%) whereas smaller numbers listed 1+  anxiolytic (38.9%) and 1+ antipsychotic (38.3%). By category, lithium (*n * =  78), bupropion (*n * =  43), clonazepam (*n * =  55), and quetiapine (*n * =  68) were the medications most commonly listed by participants (i.e., mood stabilizer, antidepressant, anxiolytic and antipsychotic, respectively).

Participants were also asked to categorize each of these medications (mood stabilizers, antidepressants, anxiolytics, antipsychotics, other/specify): 96% correctly specified and categorized both mood stabilizers and anxiolytics, 97% for antidepressants, and 86% for antipsychotics. This high level of accuracy fosters confidence that participants, in fact, are persons with BD.

### BDS_x_

The Bipolar Disorder Symptom Scale (O’Rourke et al. 2016) was developed for ecological momentary sampling of BD symptoms in the moment. Based on the format of the PANAS (Positive and Negative Affect Scale; Watson et al. 1988); respondents indicate the extent to which to each of 20-items describes how they feel right now, at this moment, ranging from not at all (0), sometimes (1), to a lot (2).

Internal consistency of BDS_x_ responses by BD I outpatients was recorded as α  = 0.90 for the depression subscale (cognitive  +  somatic symptoms; Osher et al. 2020) but lower for the hypo/mania subscale at α = 0.76 (affrontive + elation/loss of insight; Kraun et al. 2020). This difference may be due to lower frequency of hypo/manic versus depressive symptoms (Judd et al. 2002).

Concurrent validity of BDS_x_ responses by BD I outpatients has been demonstrated relative to the self-reported Hamilton Rating Scale for Depression (HAMD-6) and the Altman Self-Rating Mania Scale (Kraun et al. 2020). Similarly, sensitivity (and specificity) are high for the BDS_x_ depression subscale at 88% (and 76%), respectively (Osher et al. 2020). Sensitivity is lower at 57% for the BDS_x_ mania subscale (90% specificity) but higher sensitivity than the Altman scale (vs. 43%; Osher et al. 2020).

### Analytical procedures

Separate confirmatory factor analytic (CFA) models were computed for those with BD I, BD II and BD NOS. This allowed us to replicate the 4-factor structure of BDS_x_ responses as previously reported (O’Rourke et al. 2016; O’Rourke et al. 2018). Each of the 20 items was assumed to load on its respective depression or hypo/mania factor. We assumed that the two depression factors (cognitive and affective symptoms) and the two hypo/mania factors were significantly correlated (affrontive symptoms and elation/loss of insight). Affrontive symptoms of hypo/mania factor were also assumed to correlate with both depression factors, consistent with mixed features specifier as defined by DSM 5 (American Psychiatric Association 2013). Models were computed using the maximum likelihood method of parameter estimation.

We report three goodness-of-fit-indices: an incremental, an absolute, and a parsimonious fit index. The comparative fit index (CFI) is an incremental index representing the extent to which a hypothesized model is a better fit to data than the null model. The standardized root mean square residual (SRMR) is an absolute index which represents the standardized difference between observed and predicted correlations within a hypothesized model. Finally, the root mean square error of approximation (RMSEA) is a parsimony index which represents the extent to which a hypothesized model fits data relative to the general population. Coefficient values greater than 0.94 for the CFI, and less than 0.055 for the SRMR and RMSEA, indicate good model fit (O’Rourke and Hatcher 2013).

### Comparing BD subtypes

We then conducted invariance analyses to compare BDS_x_ item responses and compared covariance between factors. Affrontive symptoms of hypo/mania were assumed to be similarly correlated with other BD factors across subtypes. Descriptive statistics were computed with SPSS v24; CFA and invariance analyses were computed with AMOS v22.

## Results

The sample was composed of 215 men and 564 women; 5 indicated another gender (e.g., transsexual). They ranged in age from 18 to 82 years (*M * =  44.48, *SD * =  13.50), with 11.75 years of education on average (SD  =  5.76). Participants lived in the U.S. (41.6%), Canada (30.7%), the U.K. (15.6%), Ireland (5%), Australia/New Zealand (3.7%), and South Africa (4%) with the remaining 3% living in ten other countries; 90% reported that they were Caucasian. See Table 1

**Table 1:** Descriptive statistics and BDS_x_ response levels by BD subtype (n  =  784)

	BD I (M, SD, α) n = 218		BD II (M, SD, α) n = 277		BD NOS (M, SD, α) n = 289	
Descriptive features						
Years of age	43.68 (13.9)		43.87 (13.1)		45.66 (13.6)	
Duration of D_x_	11.85 (11.0)*		9.72 (8.85)**		11.39 (8.89)*	
Years of education	11.89 (5.92)		12.32 (5.96)*		11.03 (5.37)*	
Comorbid D_x_	1.05 (1.37)		1.25 (1.47)		1.18 (1.38)	
Psychotropic R_x_	0.86 (1.72)*		1.16 (1.70)**		0.62 (1.16)*	
BDS_x_ response levels						
Cognitive S_x_ depression	11.94 (3.57)	.90	12.15 (3.75)	.91	12.68 (3.40)	.90
Somatic S_x_ depression	8.84 (2.59)	.88	9.27 (2.48)	.87	9.11 (2.43)	.85
Elation/loss of insight	7.21 (2.51)*	.79	6.63 (2.12)**	.73	7.32 (2.39)*	.74
Affrontive S_x_ of mania	8.20 (2.85)*	.82	8.00 (2.89)*	.85	8.81 (2.94)**	.83

*Duration of D*_*x*_ years since BD diagnosis; *Comorbid D*_*x*_ number of comorbid psychiatric conditions; *Psychotropic R*_*x*_ number of prescribed psychotropic medications

^*^Significantly differs from one other group

^**^Significantly differs from both other groups

Thirty percent of participants indicated that they were married; almost as many indicated that there were currently single (28%). A further 20% were partnered (e.g., civil union) and 18% separated or divorced. Only 2% were widowed. Slightly more men than women indicated a BD I diagnosis, χ^2^ [df  =  4]  =  9.75, *p * =  0.05.

Participants were diagnosed with BD 10.91 years ago on average (*SD * =  9.55, range 1 month–61.17 years), they had 1.17 comorbid conditions (*SD * =  1.41, range 0–6), and were currently prescribed 0.88 psychotropic medications on average (*SD * =  1.54, range 0–15). Several indicated that they no longer take psychotropic medications; instead, they self-medicate with marijuana and avoid clinical contact.

### BD subtypes

We recruited 218 participants with BD I, 277 with BD II, and 289 with BD NOS. Subtypes did not differ in participant age [*F *(2772)  =  1.74, *p * =  0.18] or number of comorbid psychiatric conditions, *F *(2781)  =  1.74, *p * =  0.18. However, those with BD II were diagnosed somewhat more recently (M  =  9.72 years) than other subtypes [*F *(2716)  =  3.34, *p * =  0.03] and were somewhat more educated that those with BD NOS, *F *(2777)  =  3.95, *p * =  0.02. Those with BD II were also prescribed more psychotropic medications than other participants, *F *(2781)  =  8.76, *p * <  0.01.

Of note, responses to depression factors did not differ across BD subtypes. And elation/loss of insight was significantly higher for those with BD I than BD II [not BD NOS; *F *(2781)  =  6.91, *p * =  0.01] as we would expect as this factor measures traditional features of mania (e.g., euphoric, talkative, invincible). By contrast, affrontive symptoms of hypo/mania were significantly higher for those with BD NOS than BD I and II, F (2781)  =  5.94, *p * <  0.01 consistent with mixed symptom presentation. See Table 1.

### 4-factor model, each BD subtype

Confirmatory factor analytic (CFA) models were computed separately to replicate the 4-factor model across BD subtypes. Each BDS_x_ item was assumed to load significantly on its respective depression or hypo/mania factor (i.e., critical ratio values  >  ∣1.96∣) and the two depression and two hypo/mania factors were assumed to be positively correlated. In accord with existing research, affrontive symptoms were also assumed also to correlate positively with both depression factors. See Fig. 1.

These findings emerged first for those with BD I, χ^2^ (*df * =  158)  =  261.74, *p * <  0.01. Goodness of fit was calculated after correcting for correlated error between 9 of 210 possible pairs. The Comparative Fit Index (CFI  ≥  0.95; CFI  =  0.95) and root mean square error of approximation were both within ideal parameters for this model (RMSEA  ≤  0.055; RMSEA  =  0.055, 0.043  <  RMSEA CL_90_  <  0.067) whereas the standardized root mean square residual (SRMR  ≤  0.010; SRMR  =  0.095) was adequate. Statistic power for this model was estimated at 0.99 (*n * =  218, *df * =  158). See Table 2.

**Table 2:** 4-factor model of BDS_x_ responses—BD I, BD II, BD NOS

BDS_x_ factors and items	BD I (*n * = 218)	BD II (*n * = 277)	BD NOS (*n * = 289)
Cognitive S_x_ of depression			
1. Miserable	0.85 (10.5)	0.87 (11.7)	0.83 (13.0)
9. Worthless	0.87 (10.8)	0.86 (11.6)	0.79 (12.3)
13. Gloomy	0.78 (9.93)	0.80 (11.0)	0.79 (12.4)
19. Hopeless	0.24 (3.44)	0.38 (5.90)^a^	0.18 (3.07)^a^
11. Insecure	0.83 (10.2)	0.76 (10.7)	0.75 (11.7)
17. Lonely	0.65 (10.5)	0.63 (11.7)	0.70 (13.0)
Somatic S_x_ of depression			
12. Fatigued	0.87 (12.6)	0.87 (15.4)	0.86 (15.0)
9. Sleepy	0.65 (11.6)	0.61 (11.7)	0.58 (9.72)
15. Exhausted	0.89 (12.8)	0.86 (15.3)	0.86 (15.0)
3. Tired	0.74 (12.6)	0.79 (15.4)	0.77 (15.0)
Affrontive S_x_ of mania			
4. Hostile	0.80 (10.5)	0.80 (14.4)	0.78 (11.6)
8. Furious	0.76 (10.3)	0.81 (11.3)	0.76 (11.3)
5. Disgusted	0.62 (8.58)	0.67 (11.5)	0.66 (9.17)
18. Suspicious	0.70 (9.20)	0.61 (10.3)	0.70 (10.5)
20. Argumentative	0.70 (10.5)	0.80 (14.4)	0.69 (11.6)
Elation/loss of insight			
6. Impulsive	0.72 (10.0)	0.83 (11.4)	0.73 (8.71)
16. Euphoric	0.80 (11.3)	0.81 (11.3)	0.78 (8.93)
10. Indestructible	0.76 (10.6)	0.69 (12.7)	0.57(9.53)
2. Talkative	0.48 (6.91)	0.42 (6.40)	0.45 (6.33)
14. Invincible	0.74 (10.0)	0.69 (11.4)	0.60 (8.71)
Goodness of fit			
RMSEA (CL_90_)	0.055 (0.043–0.067)	0.054 (0.043–0.064)	0.051 (0.041–0.061)
SRMR	0.095	0.063	0.069
CFI	0.95	0.96	0.96

^a^Significantly differs from one other group

^**^Significantly differs from both other groups

Similar results emerged for those with BD II, χ^2^ (*df * =  157)  =  281.45, *p * <  0.01. Again the CFI (0.96) and RMSEA (0.054; 0.043  <  RMSEA CL_90_  <  0.064) are within ideal parameters. The SRMR (0.063) is adequate yet statistical power is again high (*d * =  0.99; *n * =  277, *df * =  157).

Lastly, we replicated the 4-factor model with those diagnosed as BD NOS, χ^2^ (*df*  =  158)  =  278.16, *p * <  0.01. And as with other subtypes, the CFI (0.96) and RMSEA (0.051; 0.041  <  RMSEA CL_90_  <  0.061) were in ideal limits and the SRMR (0.069) was adequate; power was high; *d * =  0.99; n  =  289, df  =  158.

### Factor structure comparisons

With baseline models computed independently for those with BD I, BD II and BD NOS, we were then able to compare the 4-factor latent structure. Specifically, we compared the strength of association between factors to determine if the latent structure is consistent across BD subtypes. This was what we found. That is, both depression and both hypo/mania factors are similarly associated; moreover, covariance between affrontive symptoms of hypo/mania, cognitive and somatic symptoms of depression are is statistically indistinguishable across subtypes. In other words, affrontive symptoms appear universal to the clinical presentation of hypo/mania. Associations are similar with elation/loss of insight and depression factors (cognitive, somatic symptoms). The 4-factor model including affrontive symptoms are consistent across BD subtypes. See Table 3.

**Table 3:** 4-Factor model of BDS_x_ responses, between BD subtype comparisons

	χ^2^	df	Δχ^2^	Δdf	SRMR	CFI	RMSEA (CL_90_)
Baseline	837.02	474	–	–	0.098	0.95	0.031 (0.028–0.035)
Cognitive-somatic							
BDI–BD II	837.04	475	0.02	1	0.098	0.95	0.031 (0.028–0.035)
BDI–BD NOS	837.55	475	0.53	1	0.099	0.95	0.031 (0.028–0.035)
BDII–BD NOS	837.42	475	0.4	1	0.098	0.95	0.031 (0.028–0.035)
BD I, BD II, NOS	837.68	476	0.66	2	0.098	0.95	0.031 (0.028–0.035)
Elation-affrontive							
BDI–BD II	839.94	477	2.26	1	0.098	0.95	0.031 (0.028–0.035)
BDI–BD NOS	838.86	477	1.18	1	0.099	0.95	0.031 (0.028–0.035)
BDII–BD NOS	837.88	477	0.2	1	0.098	0.95	0.031 (0.028–0.035)
BD I, BD II, NOS	840.05	478	2.37	2	0.098	0.95	0.031 (0.028–0.035)
Cognitive-affrontive							
BDI–BD II	840.06	479	0.01	1	0.098	0.95	0.031 (0.028–0.035)
BDI–BD NOS	840.09	479	0.04	1	0.098	0.95	0.031 (0.028–0.035)
BDII–BD NOS	840.17	479	0.12	1	0.098	0.95	0.031 (0.028–0.035)
BD I, BD II, NOS	840.18	480	0.13	2	0.098	0.96	0.031 (0.027–0.034)
Somatic-affrontive							
BDI–BD II	840.22	481	0.04	1	0.097	0.96	0.031 (0.027–0.034)
BDI–BD NOS	840.26	481	0.08	1	0.098	0.96	0.031 (0.027–0.034)
BDII–BD NOS	840.43	481	0.25	1	0.098	0.96	0.031 (0.027–0.034)
BD I, BD II, NOS	840.45	482	0.27	2	0.098	0.96	0.031 (0.027–0.034)

### BDS_x_ item consistency

Lastly we compared items responses across BD subtypes to examine the psychometric properties of BDS_x_ scale responses. Responses were equivalent for 19 of 20 items across BD subtypes; only responses to the ‘hopeless’ item significantly differed. More precisely, those with BD II reported significantly higher hopelessness than those with BD NOS (β  =  0.38 vs. β  =  0.18). See Table 2. This finding is noteworthy as hopelessness is associated with suicide risk, suggesting that participants with BD II may be at greater risk of self-harm (Neufeld and O’Rourke 2009). See Table 4.

**Table 4:** BDS_x_ item comparisons—BD I, BD II, BD NOS

	χ^2^	df	Δχ^2^	Δdf	SRMR	CFI	RMSEA (CL_90_)
Cognitive S_x_ of depression							
1. Miserable	842.46	484	2.01	2	0.098	0.96	0.031 (0.028–0.035)
9. Worthless	846.45	486	3.99	2	0.098	0.95	0.031 (0.028–0.035)
13. Gloomy	846.48	488	0.03	2	0.098	0.96	0.031 (0.027–0.034)
19. Hopeless							
BDI–BD II	850.03	489	3.55	1	0.098	0.95	0.031 (0.028–0.035)
BDI–BD NOS	846.57	489	0.09	1	0.098	0.95	0.031 (0.028–0.035)
BDII–BD NOS	851.85	489	5.37*	1	0.098	0.95	0.031 (0.028–0.035)
BD I, BD II, NOS	852.69	490	6.21*	2	0.098	0.95	0.031 (0.028–0.035)
11. Insecure	853.65	492	0.96	2	0.098	0.95	0.031 (0.027–0.034)
17. Lonely	853.65	492	1.64	2	0.098	0.95	0.031 (0.027–0.034)
Somatic S_x_ of depression							
12. Fatigued	853.83	494	0.18	2	0.098	0.96	0.031 (0.028–0.035)
9. Sleepy	854.16	496	0.33	2	0.097	0.96	0.030 (0.027–0.034)
15. Exhausted	856.24	498	2.08	2	0.097	0.96	0.030 (0.027–0.034)
3. Tired	856.24	498	0.37	2	0.097	0.96	0.030 (0.027–0.034)
Affrontive S_x_ of mania							
4. Hostile	860.07	500	3.83	2	0.097	0.96	0.030 (0.027–0.034)
8. Furious	860.75	502	0.68	2	0.097	0.96	0.030 (0.027–0.034)
5. Disgusted	861.1	504	0.35	2	0.097	0.96	0.030 (0.027–0.034)
18. Suspicious	866.67	506	5.57	2	0.097	0.95	0.030 (0.027–0.034)
20. Argumentative	866.67	506	2.84	2	0.097	0.95	0.030 (0.027–0.034)
Elation/loss of Insight							
6. Impulsive	867.55	508	0.88	2	0.097	0.96	0.030 (0.027–0.033)
16. Euphoric	869.23	510	1.68	2	0.098	0.96	0.030 (0.027–0.033)
10. Indestructible	871.83	512	2.6	2	0.098	0.96	0.030 (0.027–0.033)
2. Talkative	873.02	514	0.27	2	0.098	0.96	0.030 (0.027–0.033)
14. Invincible	840.45	514	1.19	2	0.098	0.96	0.030 (0.027–0.033)

## Discussion

The results of this study provide further support for the 4-factor model of BD symptomology. This model was replicated independently with BD I, BD II and BD NOS participants in which affrontive symptoms of hypo/mania are correlated with elation/loss of insight and both depression factors. This presentation of depressive symptomology as correlated cognitive and somatic factors is consistent with unipolar depression research (Ward 2006).

Less consistent with existing research is our result suggesting that affrontive symptoms are central to the clinical presentation of hypo/mania across BD subtypes. Unlike elation/loss of insight, this facet of hypo/mania is correlated with both cognitive and somatic factors, which indicates ways in which mixed symptomology can present. As we noted, affrontive symptoms were significantly greater for those with BD NOS compared to participants with BD I and BD II.

Our findings also provide psychometric support for the BDS_x_ across BD subtypes. As we noted, responses to 19 of 20 items were equivalent across subtypes; only responses to the ‘hopeless’ item were significantly greater by those with BD II. For a separate study (O’Rourke et al. 2018), participants 50+  years of age were later asked if they had made one or more suicide attempts (*n * =  103): 43% and 45% of BD NOS and BD I reported a prior attempt (15 of 35, and 10 of 22, respectively); by contrast, the majority of participants with BD II reported one or more attempt (28 of 46, or 61%). While these percentages apply only to older participants who agreed to follow up contact, they are consistent with our finding that those with BD II reported feeling more hopeless than other participants. This finding appears to differ from other research showing no difference in suicide risk by BD subtype (Tondo et al. 2016; Undurraga et al. 2011). Further study is warranted.

### Limitations and future research

The results of this study suggest that the 4-factor model of symptomology applies to those with BD I, BD II and BD NOS. This includes affrontive symptoms of hypo/mania. These results need to be replicated with those with cyclothymia and participants diagnosed in accord with DSM 5 or ICD 10 criteria.

We recruited a large sample allowing us to compare BD subtypes. This was achieved by recruiting an international sample of adults with BD. Social media recruitment remains a somewhat novel methodology but is especially well-suited to low prevalence populations like BD (King et al. 2014). Notably, this also enabled us to recruit participants who avoid clinical contact in contrast to most BD research.

International data collection also allowed us to (somewhat) address a fundamental measurement challenge with BD research. Specifically, most with BD are euthymic most of the time, depressed sometimes and hypo/manic sporadically (Saunders and Goodwin 2010). As reported by Osher et al. (2020), just 16% of BD outpatients presented as depressed and 14% with hypo/mania at routine biannual clinical visits over 6-months. In other words, without large samples, it is unlikely to have sufficient numbers to identify and measure hypo/mania. It is therefore easy to underreport the psychometric efficacy of hypo/mania in small, mostly asymptomatic BD samples. Differences in internal consistency for the depression versus hypo/mania subscale may be due to lower overall frequency of hypo/mania in this and most BD outpatient samples.

These findings contribute to a growing body of research pointing to the existence of an interpersonal, aggressive cluster of hypo/mania symptoms unacknowledged in current diagnostic criteria. Our results suggest that these symptoms are consistent across subtypes and correlated with all other BD factors. Further research is needed with inpatient samples and participants recruited using more traditional means (vs. mood disorders clinic patients).

## Data Availability

Anonymized data are available from the corresponding author on request.
